# The choice of an autocorrelation length in dark-field lung imaging

**DOI:** 10.1038/s41598-023-29762-y

**Published:** 2023-02-15

**Authors:** Simon Spindler, Dominik Etter, Michał Rawlik, Maxim Polikarpov, Lucia Romano, Zhitian Shi, Konstantins Jefimovs, Zhentian Wang, Marco Stampanoni

**Affiliations:** 1 Swiss Light Source, Paul Scherrer Institute, 5232 Villigen, Switzerland; 2 Institute for Biomedical Engineering, ETH Zürich, 8092 Zürich, Switzerland; 3 Department of Engineering Physics, Tsinghua University, Haidian District, 100080 Beijing, China; 4 Key Laboratory of Particle & Radiation Imaging, (Tsinghua University) Ministry of Education, Haidian District, 100080 Beijing, China

**Keywords:** Biomedical engineering, Translational research, Imaging and sensing, Medical imaging, Chronic obstructive pulmonary disease

## Abstract

Respiratory diseases are one of the most common causes of death, and their early detection is crucial for prompt treatment. X-ray dark-field radiography (XDFR) is a promising tool to image objects with unresolved micro-structures such as lungs. Using Talbot-Lau XDFR, we imaged inflated porcine lungs together with Polymethylmethacrylat (PMMA) microspheres (in air) of diameter sizes between 20 and 500 $\upmu \hbox {m}$ over an autocorrelation range of 0.8–5.2 $\upmu \hbox {m}$. The results indicate that the dark-field extinction coefficient of porcine lungs is similar to that of densely-packed PMMA spheres with diameter of ${200}\,\upmu \hbox {m}$, which is approximately the mean alveolar structure size. We evaluated that, in our case, the autocorrelation length would have to be limited to ${0.57}\,\upmu \hbox {m}$ in order to image ${20}\,\hbox {cm}$-thick lung tissue without critical visibility reduction (signal saturation). We identify the autocorrelation length to be the critical parameter of an interferometer that allows to avoid signal saturation in clinical lung dark-field imaging.

## Introduction

Emphysema and fibrosis are irreversible changes of the lungs micro-structure, which reduce the oxygen intake. They are a common result of chronic lung illnesses like the chronic obstructive pulmonary disease (COPD), one of the leading causes of death globally^1,2^. The trigger for COPD in particular can be attributed in large parts to smoking^3^, air pollution^4^ and toxic fumes^5^ in the working environment. Early detection of chronic lung diseases is a necessity to prevent a reduced quality of life and death for millions of people.

A promising candidate for early detection of emphysema and fibrosis is X-ray dark-field chest imaging. While conventional X-ray imaging relies only on the attenuation of X-rays by the tissue, imaging relying on refraction has attracted a lot of attention in the last decade. Imprinting an intensity pattern into an X-ray beam makes it possible to observe how it is deformed or diffused. The deformation due to refraction on structures large enough to be directly resolved by the imaging system leads to phase-contrast imaging^6–8^, whose use is explored in areas like mammography^9^, intraoperative breast CT^10^ or virtual histopathology^11,12^. We focus on the diffusion, referred to as X-ray Dark-Field Radiography (XDFR), which occurs due to multiple deflections on smaller structures, for example when the beam refracts on the many tissue-air interfaces of a lung. While a high density of tissue-air interfaces in a healthy lung strongly diffuses an X-ray beam, as strong dark-field signal, lung tissue with pathologies affected by emphysema or fibrosis^13^ diffuses less. Multiple studies have already shown that XDFR, in comparison with established methods like computed tomography, can provide better contrast for emphysema with a fraction of the dose^13,14^. The three-grating Talbot-Lau interferometer is a particular implementation of XDFR that gains traction in the medical imaging realm. It uses two absorption gratings and a phase grating to create an interference pattern from an incoherent X-ray tube, the analysis of which yields the three contrasts: conventional absorption, phase (resolvable refraction) and dark field (DF) (diffusion). The development started by successfully analysing murine lungs^15–17^, studies with porcine lungs^18,19^ and deceased human bodies^20^. Recently clinical human studies^14,21,22^ of chest radiography have been published, as well as a first design for a dark-field CT system^23,24^, which shows the diagnostic potential^25^ that dark-field lung imaging provides.

**Figure 1:**
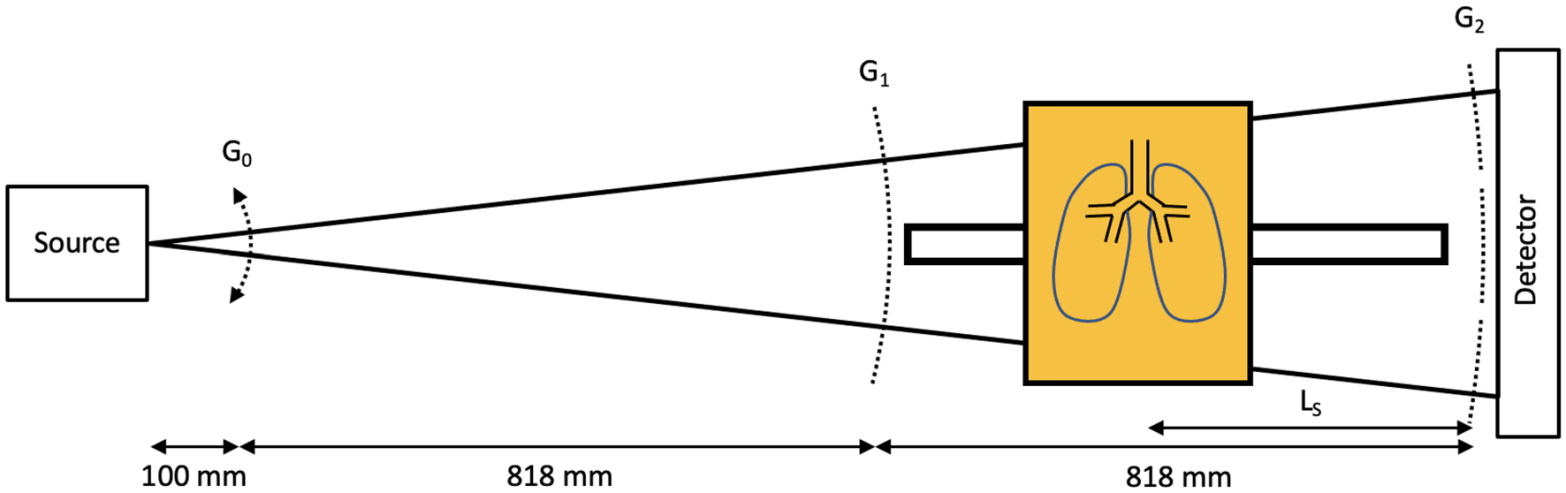
Sketch of a symmetric Talbot-Lau interferometer as used for this study. The system contains a single-tile source grating $\text {G}_0$ that is used for phase stepping, a single tile phase grating $\text {G}_1$ and a stitched three tile analyser grating $\text {G}_2$. In between $\text {G}_1$ and $\text {G}_2$ a rail with a sample holder is mounted to manually move the sample holder along the beam axis.

The object-induced diffusion of the Talbot carpet is caused by many interfaces of refractive-index changes^26^. This has been the topic of multiple studies that quantitatively model and simulate the emergence of the dark-field signal in, most often, distributions of ideal spheres^27,28^. Those studies identify the autocorrelation length as the crucial parameter determining the sensitivity of an interferometer to the physical feature size. For Talbot-Lau interferometers it is defined as^27^

1 $$\begin{aligned} \xi = \frac{\lambda L_s}{p_2}\, \end{aligned}$$

where $\lambda$ is the wavelength of the X-rays, $L_s$ is the distance of the sample to $\textrm{G}_2$ and $p_2$ is the periodicity of the imprinted pattern (the period of the $\textrm{G}_2$ grating as described in Fig. 1). Analytical theoretical frameworks exist for spheres and allow to fit data of more general shapes^29^. However, they fall short of interpreting more complicated objects and poly-chromatic setups^30^ or predicting measurements of complex samples through simulations. More realistic calculations resolve to numerical propagation of the wave front and emphasise the impracticality of interpreting the strength of the dark-field signal as a measure of the real-space autocorrelation function^30^.

So far only a few experimental points of the dark-field signal’s dependence on the autocorrelation length for lungs were reported^31^.

In the design of a dark-field imaging system the autocorrelation length plays a crucial role. It defines, together with the energy used^26^, the amount of diffusion caused by structures of a particular size. A given system is most sensitive to structures of approximately the size equal to its autocorrelation length^27^. Therefore, adjusting the autocorrelation length of the system allows to optimise the amount of diffusion for a defined structure size. It might seem natural to design a lung XDFR system with the autocorrelation length on the scale of the alveoli, around ${250}\,\upmu \hbox {m}$. However, it is impractical. Firstly, for energies in the ${40}-{70}\,\hbox {keV}$ range, which are necessary to penetrate a human chest, and a system length of a few meters, it would require grating structures with extremely high aspect ratios (below ${0.1}\,\upmu \hbox {m}$ pitch at structure heights of around ${200}\,\upmu \hbox {m}$), impossible to manufacture with the current technology. Secondly, even if such gratings would be available, a prohibitively large dose would be necessary such that that the extremely diffused pattern can still be resolved. The choice of the suitable parameters for a lung X-ray dark-field radiography system thus requires the knowledge of the sensitivity of the dark-field signal to the microstructure of the lung tissue over a range of the autocorrelation lengths.

In this work, we measured the strength of the dark-field signal of porcine lungs over a broad range, ${0.8}-{5.2}\,\upmu \hbox {m}$, of autocorrelation lengths. Porcine lungs are generally a good stand-in for human lungs, and have been used extensively in translational medicine^32^. Even though they show slightly different anatomical features in terms of number and placement of lung lobes compared to human lungs, the alveolar size in pig and humans is considered comparable, while porcine lungs might show slightly smaller features^33–35^. The lungs were passively inflated in a purposely built vacuum chamber and imaged alongside PMMA microspheres. Our results show that the relative strength of the dark-field signal of the lung tissue is comparable to that of a similar thickness of densely packed ${200}\,\upmu \hbox {m}$–diameter PMMA spheres in the whole investigated range of the autocorrelation length. Finally, we elaborate on the potential consequences of our results for an informed choice of an autocorrelation length for the design of a human lung XDFR imaging system.

## Methods

### Inflation by negative pressure

**Figure 2:**
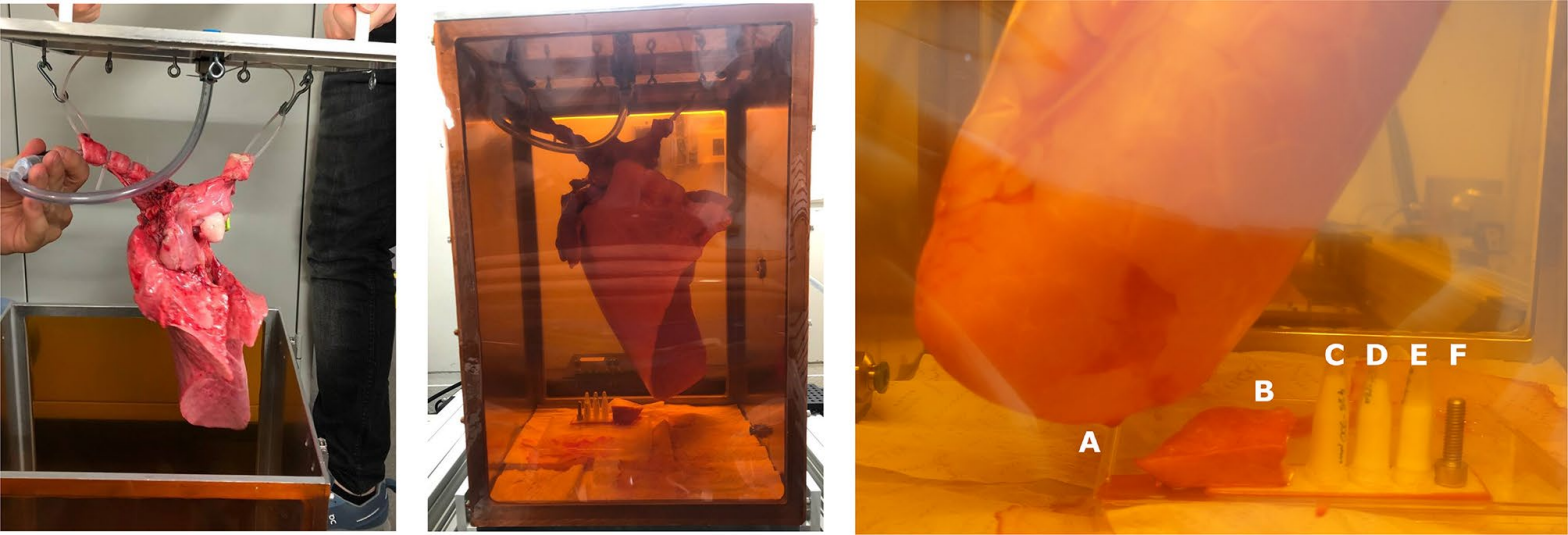
Mounting of a left porcine lung for imaging. *Left:* The right main bronchus is sealed off by a clamp (green) and fixed to the lid of the vacuum chamber with zip ties. The tube is securely fixed into the trachea and connected to the inlet in the lid. *Middle:* The left porcine lung inflated inside the vacuum chamber at −30 mbar. *Right:* Point of view from $\hbox {G}_2$ of the symmetric TLGI into the vacuum chamber. (A) Lateral basal segment of the inflated left porcine lung. (B) A piece of non-inflated porcine lung tissue. (C), (D), (E) Eppendorf tubes containing PMMA spheres of 425–500 $\upmu \hbox {m}$, 180–212 $\upmu \hbox {m}$ and 20–27 $\upmu \hbox {m}$ diameter, respectively. (**F**) A M4 steel screw (${4}\,\hbox {mm}$ in diameter) as reference marker.

Under *in vivo* conditions the lungs are inflated by a reduced surrounding pressure forcing air into the lungs. Inspired by^36^, we designed and built a vacuum chamber shown in Fig. 2. Lungs fitted with a tracheal tube can be hung in the chamber and connected to the surrounding atmosphere via an inlet through its lid. Once the lid was sealed and secured an external pump built up a negative pressure inside the chamber. The pressure was monitored via an absolute pressure sensor (PCR 280, Pfeiffer Vacuum GmbH, Asslar, Germany) and controlled via a valve. Two opposite walls of the chamber had large ${0.075}\,\hbox {mm}$–thick Kapton-foil windows (Dupont, Wilmington, DE, USA). The foil was glued to the aluminium frame with Araldite Standard two-component glue (Huntsman Corp., The Woodlands, TX, USA).

#### Porcine lungs ethics approval

Fresh porcine lungs were ordered through the veterinary department and obtained directly from the slaughterhouse (Schlachtbetrieb Zürich AG) of Zurich, Switzerland. As the pigs in question were destined to be slaughtered regardless of our study, no further ethics approval was necessary. In Switzerland, lungs fall into the category “K1” and are thus considered “risk materials”. Materials of category “K1” are banned from entering the food-cycle and have to be incinerated. This was ensured by disposing the samples at the cadaver collection facility after experiments, provided by each municipality in Switzerland. Throughout the study 15 porcine lungs have been acquired.

### Imaging system

The measurements for the study have been done using a symmetric wide-field-of-view cone-beam grating interferometer schematically depicted in Fig. 1. A Talbot-Lau grating interferometer is a technical solution to perform X-ray interferometry with incoherent sources and pixel sizes much larger than the fringes. It consists of two absorption gratings, $\text {G}_0$ and $\text {G}_2$, as well as a phase-shifting grating $\text {G}_1$. $\text {G}_0$ acts as an array of narrow sources for partial coherence^6^, $\text {G}_2$ with the same periodicity as the incident fringe allows to measure the intensity profile of the Talbot carpet that is created by the phase grating $\text {G}_1$. A Talbot-Lau interferometer provides access to the absorption, refraction and small-angle-scattering properties of the sample. The corresponding signals are obtained by stepping one of the gratings and observing the intensity of the fringe pattern analysed by $\text {G}_2$ for each pixel. The signal retrieval is discussed in the section “Image analysis”.

Symmetric geometries place $\text {G}_1$ in the center between $\text {G}_0$ and $\text {G}_2$ and have the advantage that all gratings have the same period^37,38^. The design energy of the setup, one for which $\text {G}_1$ shifts the phase of the wavefront by $\pi$ and for which the distances between the gratings have been chosen, was ${46}\,\hbox {keV}$ and the total length was ${1766}\,\hbox {mm}$. $\text {G}_0$, $\text {G}_1$ and $\text {G}_2$ were placed at ${100}\,\hbox {mm}$, ${918}\,\hbox {mm}$ and ${1736}\,\hbox {mm}$ away from the focal spot of the x-ray source, respectively. The detector was placed ${3}\,\hbox {cm}$ downstream of $\text {G}_2$. This allows to move samples between $\text {G}_1$ and $\text {G}_2$ for a $L_s$-range of roughly ${75}-{760}\,\hbox {mm}$ and a range of possible autocorrelation lengths between ${0.5}-{5.2}\,\upmu \hbox {m}$ for an effective energy^39^ of 46 keV.

$\text {G}_0$ and $\text {G}_2$ have a period of ${4.2}\,\upmu \hbox {m}$ at ${180}\,\upmu \hbox {m}$ height. They are gold-plated absorption gratings made by the LIGA process^40^ on graphite substrates manufactured by Microworks (Karlsruhe, DE). In total three $80\,\times \,60\hbox {mm}^{2}$ tiles build up $\text {G}_2$, while a single tile $\text {G}_0$ is used for phase-stepping. The phase-stepping was done with a P-841.6 piezoelectric-motor (Physik Instrumente (Karlsruhe, DE).

The phase grating $\text {G}_1$ was fabricated on a ${300}\,\upmu \hbox {m}$ thick N-type $<100>$ double side polished 8-inch silicon wafer. First, the wafer was coated with a layer of MEGAPOSIT SPR220-3.0 positive tone photoresist and patterned using Heidelberg DWL66+ direct laser writer tool. The photoresist pattern was used as an etching mask for the following Si deep reactive ion etching process in a SPTS Rapier etching system. The process was optimized to ensure uniform etching depth and vertical trench sidewalls according to a previous report^41^. The grating lines were etched into the silicon substrate to a depth of ${59}\,\upmu \hbox {m}$ for a $\pi$-shift at ${46}\,\hbox {keV}$. Finally, a single tiled $\text {G}_1$ grating was diced out from the wafer to a size of $160\,\times \,18\,\hbox {mm}^{2}$.

All gratings were bent around the source so that the lines were parallel to the beam across the whole wide field of view. The X-Ray source used was a Comet MXR-225HP/11 (Comet Group, Flamatt, CH) with a focal spot size of ${0.4}\,\hbox {mm}$ and was operated with 70 kVp at ${10}\,\hbox {mA}$. For detection a prototype detector by DECTRIS (Baden-Daettwil, CH) was used. This detector is a direct-conversion photon-counting device with a ${750}\,\upmu \hbox {m}$–thick Cadmium-Telluride layer as the active medium and with an area of 256 $\times$ 3096 isotropic ${75}\,\upmu \hbox {m}$ pixels. A single energy threshold of 20 keV was used for all measurements. The spectrum was filtered with a ${3}\,\hbox {mm}$–thick aluminium filter placed between the source and $\text {G}_0$. The filtered spectrum has its effective energy at roughly 46 keV, the design energy of $\text {G}_1$. The sample was placed between $\text {G}_1$ and $\text {G}_2$ on a vertical-translation stage. The lung experimental chamber was designed to generously accommodate a fully inflated lung, without the latter touching the chamber’s walls. This resulted in the autocorrelation being limited to a range between ${0.8}-{3.6}\,\upmu \hbox {m}$ as it was physically impossible to place the lung very close to the gratings.

**Figure 3:**
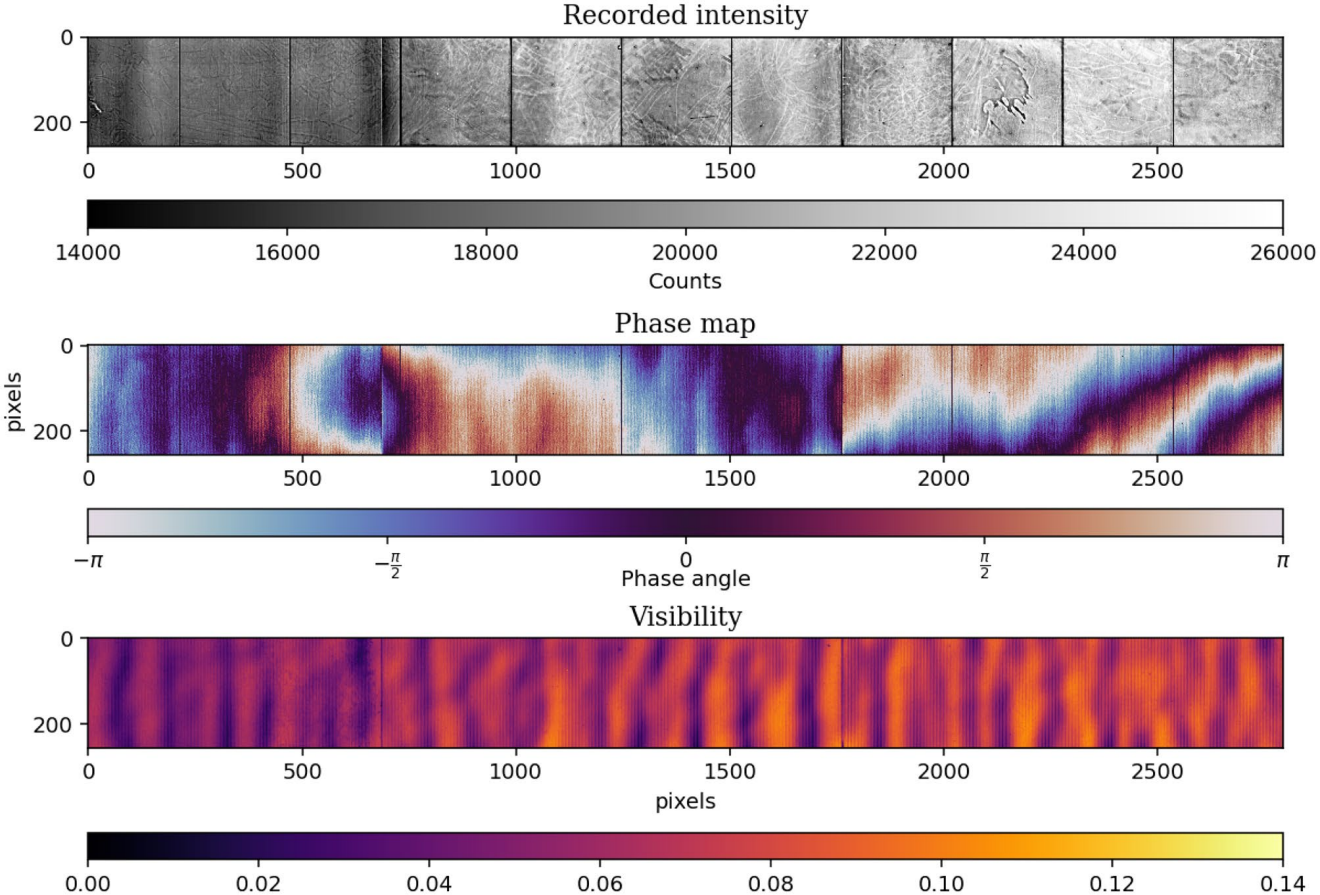
Reference phase-stepping scan without the sample in the beam. *Top:* The intensity profile. The detector is structured in 256 $\times$ 256 pixel sub-units with a two pixel gap in-between. The structures on the active area result from the Cadmium-Telluride crystals of the detector as well as the gratings gaps of $\text {G}_2$ in the beam (well visible on the phase map as sudden value shifts). A flat-field correction removes these features from the image. *Center:* The phase profile of the system. The phase is the offset of the phase stepping curve as described in Eq. (2). It is tuned to be as homogeneous as possible by aligning the gratings. *Bottom:* The visibility profile. The visibility is also obtained from Eq. (2) and characterizes the coherence of the system.

A reference phase-stepping scan without the sample in the beam is shown in Fig. 3. The intensity map is shown at the top, the phase map in the middle and the visibility map at the bottom. The average visibility in the field of view is ${6}\,\%$, which is largely due to local defects of the absorption gratings^42^, which appear as dark areas on the visibility map. The setup is optimized to have a high angular sensitivity and it is aligned to our best capabilities as the rather uniform phase map suggests.

#### PMMA microspheres

Three sizes of PMMA microspheres samples were obtained from Cospheric LLC (Santa Barbara, California 93160, USA): ${20}-{27}\,\upmu \hbox {m}$, ${180}-{212}\,\upmu \hbox {m}$ and ${425}-{500}\,\upmu \hbox {m}$. The diameters were chosen to cover the structure size distribution of alveoli in lungs. PMMA was the material of choice as it features an attenuation coefficient similar to lung-tissue.

The diffusion induced by a sample depends on the number of interfaces. The PMMA spheres have a similar attenuation coefficient as tissue but have air gaps surrounding the spheres while lung tissue surrounds its air spaces. To estimate a comparable sample thickness of a lung’s region of interest and PMMA spheres, the air-PMMA volume filling is inverted to calculate the attenuation (Eq. 3) and R-values (Eq. 5). This allows a comparison between microspheres and lung tissue of roughly the same thickness by selecting a region of interest through the absorption value. The correction was performed by assuming an average mono-sphere packing volume fraction of ${64}\,\%$^43^. This leads to an inversion factor of 1.778 = 0.64/(1-0.64).

### Measurement protocol

Lungs were hung in the inflation chamber as shown in Fig 2. Alongside the inflated lung we imaged a frozen piece of porcine lung tissue, three Eppendorf tubes filled with microspheres of the size ranges 20–28 $\upmu \hbox {m}$, 180–212 $\upmu \hbox {m}$ and 425–500 $\upmu \hbox {m}$ and an M4 screw were used.

The chamber was mounted on a vertical stage to measure the sample at different heights. Reference images were taken with the beam going through a ${5}\,\hbox {cm}$–high window between the chamber and the stage. The vertical axis was mounted to the side of an X95 aluminum profile perpendicular to the optical axis. This allowed to move the lung container over rails to the desired position between $\text {G}_1$ and $\text {G}_2$. The pressure sensor and the vacuum pump were attached to the inflation chamber and a constant negative pressure of ${30}\,\hbox {mbar}$ was applied to inflate the lungs.

For every autocorrelation length, the image acquisition always started with a reference measurement followed by a series of sample projections with a ${8}\,\hbox {mm}$ vertical movement in between. At every sample position the exposure time was ${25}\,\hbox {s}$ in total, distributed evenly over a series of five phase-stepping projections with a G0 movement of ${0.84}\,\upmu \hbox {m}$ between each image. In order to change the autocorrelation length, the aluminium profile with the sample stage was moved manually along the optical axis in steps of ${2}\,\hbox {cm}$. A separate measurement of frozen porcine lungs and PMMA microspheres outside the inflation chamber allowed a closer positioning to $\text {G}_1$ and $\text {G}_2$ and thus a wider range of the autocorrelation lenghts.

#### Image analysis

The signal-retrieval was performed with linear least-squares fit to every *i*-th pixel with the model^44^

2 $$\begin{aligned} I^i_x = \frac{I^i}{2} \left( V^i \ \sin \left( \frac{2 \pi x}{p_{\textrm{G}0}} + \phi ^i \right) + 1 \right) \, \end{aligned}$$

where the intensity measured at the $\text {G}_0$ in the position *x* is modelled by the intensity of the beam $I^i$, visibility of the phase-stepping curve $V^i$ and the phase-shift $\phi ^i$, $p_{\textrm{G}_0}$ is the period of the $\text {G}_0$ grating. The decrease, and the attenuation image $\Gamma ^i$ was obtained by relating $I^i$ to the measured reference intensity image $I^i_0$ (without the object in the beam):

3 $$\begin{aligned} \Gamma ^i = -\ln \left( \frac{I^i}{I_0^i}\right) . \end{aligned}$$

The dark-field signal is defined in a similar manner to the Beer-Lambert law as an exponential decrease of the visibility of the interference pattern per thickness and is also corrected with a flat-field value:

4 $$\begin{aligned} \Sigma ^i = -\ln \left( \frac{V^i}{V_0^i}\right) \end{aligned}$$

where $\Sigma$ is the dark-field signal and $V_0$ the visibility of the flat-field. The R value is the ratio of the dark-field and the absorption, and gives a thickness-normalized value of dark-field signal, or the average dark-field extinction coefficient, in the case of homogeneous objects^45^:

5 $$\begin{aligned} R^i = \frac{\Sigma ^i}{\Gamma ^i}. \end{aligned}$$

The images were corrected for beam hardening^46^ through phase-stepping measurements of flat and solid PMMA plates, spanning the full field of view, with thicknesses between ${1}-{18}\,\hbox {cm}$, revealing the non-linearity of the attenuation signal. We pragmatically modelled the attenuation curve with a quadratic polynomial, and the change in the dark-field signal with a first-degree polynomial. More details on the beam hardening correction are presented in the Supplementary Information.

Next, the images were stitched together vertically. The overlapping part was averaged.

The R-value was displayed after median filter (radius of 6 pixels) to remove large values from the background from dividing values close to zero. The regions of interest were manually located throughout the different autocorrelation lengths to match the same spot as good as possible, while the radius was adjusted to account for the magnification changes.

We introduced a *saturation score* to account for the saturation of the dark-field signal, which occurs when there is no measurable visibility left after the sample. For each *i*-th pixel it compares the amplitude in the intensity variation during phase-stepping to the Poisson-distributed quantum noise:

6 $$\begin{aligned} S_{sat}^i = \frac{\Sigma (I^i_{max})}{0.5(I^i_{max} - I^i_{min})} = \frac{2\sqrt{I_{max}^i}}{I_{max}^i - I_{min}^i} = \frac{2\sqrt{I_{max}^i}}{V_0^i(I_{max}^i + I_{min}^i)} \, \end{aligned}$$

where the max and min are taken over the phase-stepping series. In the last step the relation $V_0^i$ = ($I_{max}-I_{min}$)/($I_{max}+I_{min}$) was inserted (this is only valid if the unit of the intensities is counts). A saturation score above one provides the information that the statistical noise in the measurement is higher than the remaining visibility amplitude. We would like to note that an increase of the visibility would always increase the denominator more than the numerator and lead to a reduced saturation score.

Similarly, the image noise defines the visibility that can be obtained even in absence of any signal. In these low signal conditions the noise can be described as^47^

7 $$\begin{aligned} \sigma _{\Sigma }^i(n, I_0^i, t^i) = \frac{\sqrt{\pi }}{\sqrt{nI_0^ie^{-\mu t^i}}}, \end{aligned}$$

where *n* is the number of phase steps, $I_0$ is the number of photons in absence of a sample, *t* and $\mu$ are thickness and the attenuation coefficient of the sample respectively. To arrive there the Beer-Lambert law was used to express *I* in terms of the incident number of photons and the thickness of the sample.

## Results

**Figure 4:**
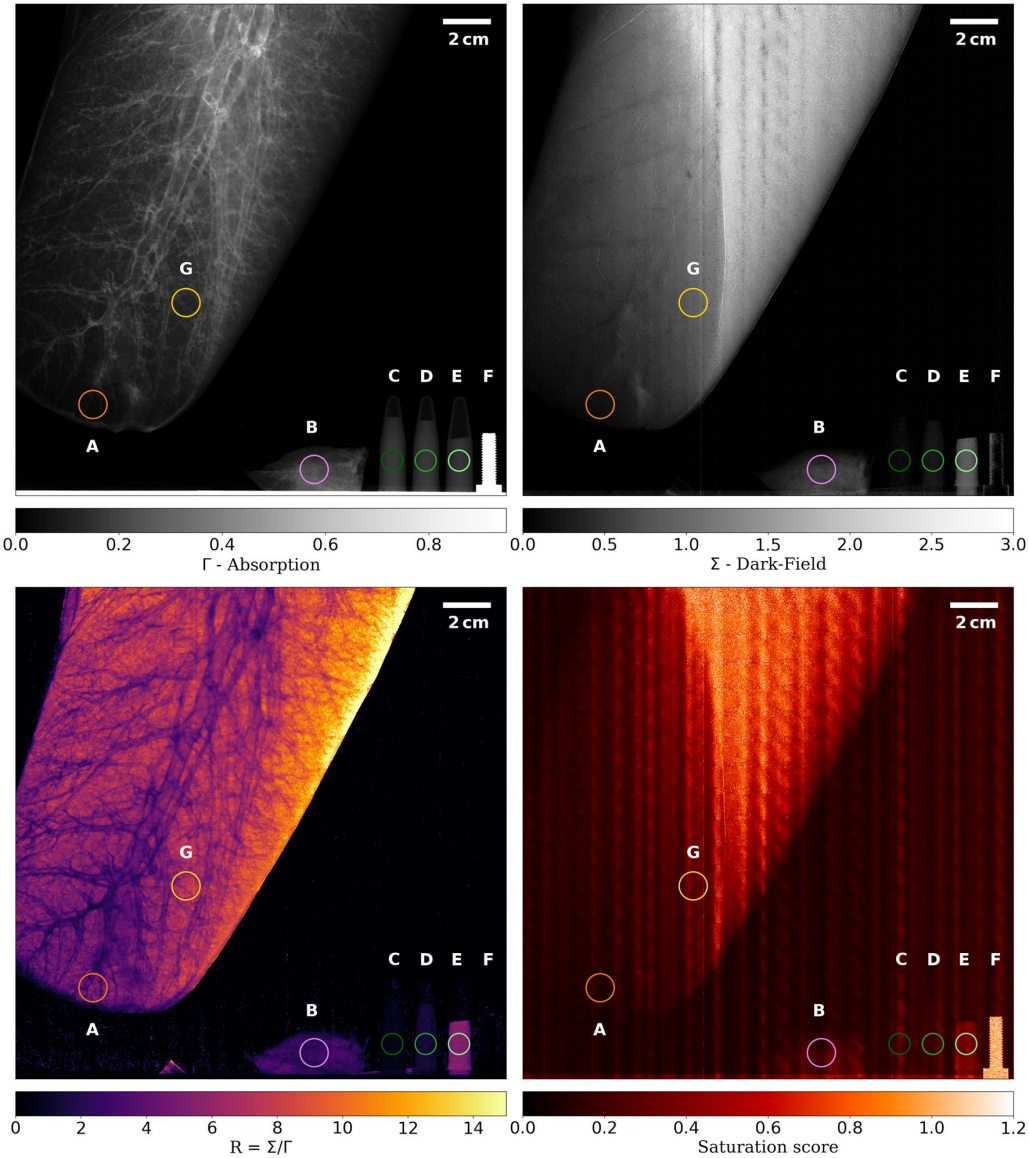
Analysed data from imaging done with sample shown in Fig. 2 at an autocorrelation length of $\xi =$ ${0.8}\,\upmu \hbox {m}$. The displayed circles mark the regions of interest that are displayed in Fig. 5. *Top-left:* absorption, *top-right:* dark-field, *bottom-left:* R-value and *bottom-right:* saturation score. (A) Lateral basal segment of the inflated left porcine lung (’Lung Inflated 1’). (B) A piece of non-inflated porcine lung tissue (’Lung Uninflated 1’). (C), (D), (E) Eppendorf tubes containing PMMA spheres of 425–500, 180–212 and 20–$27\,\,\upmu \hbox {m}$ diameter, respectively. (F) A M4 steel screw (${4}\,\hbox {mm}$ in diameter) as reference marker. G) thicker segment of inflated porcine lung (’Lung Inflated 2’).

**Figure 5:**
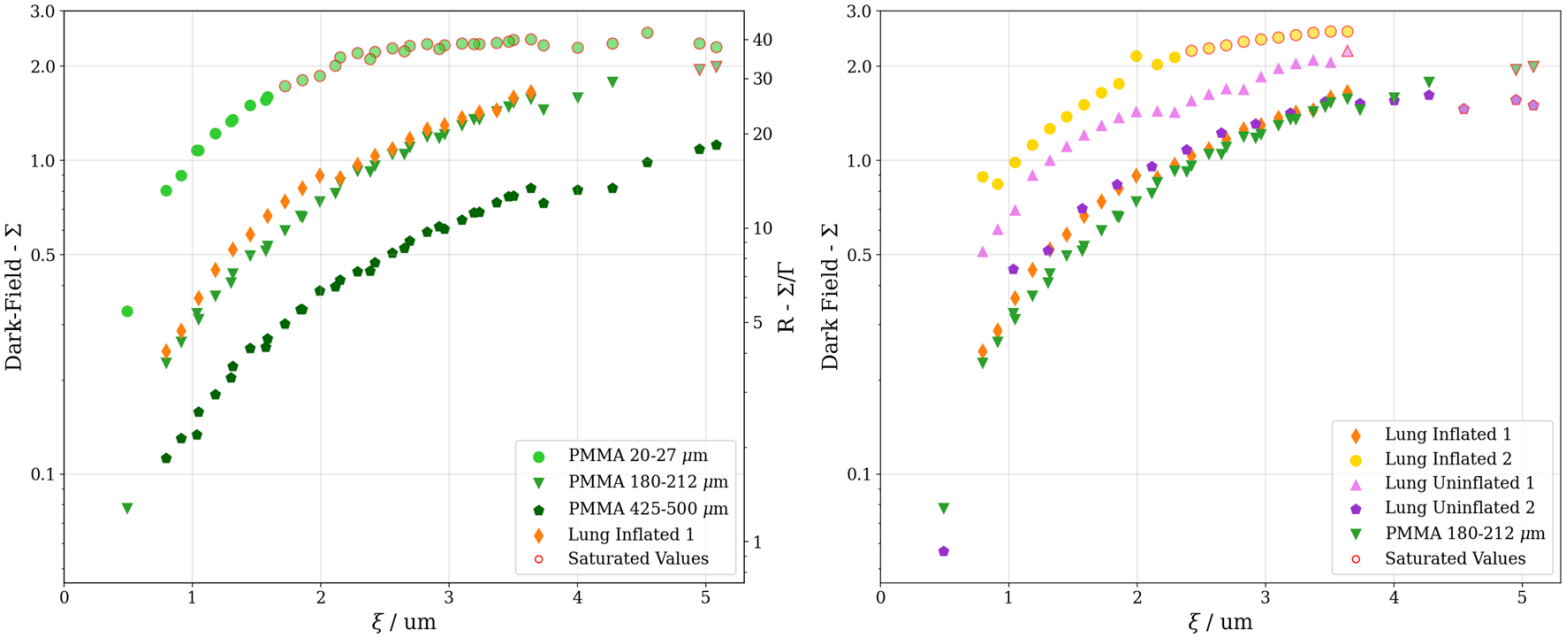
Dark-field signal against autocorrelation length. For the sake of clarity, measurements series have been grouped appropriately and plotted separately. *Left:* Data obtained from the thickness-adjusted PMMA spheres taken from the circles labeled ’C’, ’D’, ’E’ on Fig. 4, as well as the non-saturating series of inflated porcine lungs ’Lung Inflated 1’ labeled with ’A’. The thickness was chosen to be the same for all regions of interest to compare the scattering of a similar amount of interfaces and to simultaneously plot the dark-field and R-value. The points with the saturation score larger than 0.78 are marked with a red outline. *Right:* Data obtained from porcine lung tissue with different thicknesses as well as the series of the ${180}-{212}\,\upmu \hbox {m}$ PMMA spheres for comparison. The inflated series ’1’ and ’2’ correspond to the regions ’A’ and ’G’ in Fig. 4. The uninflated series ’1’ corresponds to region ’B’ in Fig. 4 while ’2’ was another different sized, not displayed piece of lung. Due to hardware limitations it was necessary to remount the sample holder at roughly ${2}\,\upmu \hbox {m}$ autocorrelation length, which induced a small glitch in the measured values.

We imaged a sample consisting of an inflated porcine lung, a frozen piece of porcine lung tissue, three vials with PMMA spheres and a metal screw, as depicted in Fig. 2. The attenuation $\Gamma$, dark-field $\Sigma$, $R = \Sigma / \Gamma$ and the saturation-score images at the lowest autocorrelation length of ${0.8}\,\upmu \hbox {m}$ are shown in Fig. 4. Data for all autocorrelation length are aggregated in two scatter plots in Fig. 5 showing the dark-field of different regions of interest as well as an equal-thickness plot with the R-value plotted on the right axis.

To estimate the maximal sample thickness before the dark-signal saturates, the lung’s base measurements (Lung inflated 1) were evaluated. The density of the lower lung during inspiration^48^ has been shown to be $\rho _{L} = 0.154\,$g/cm$^3$. The mass attenuation coefficient of lung tissue^49^ (LT) at ${46}\,\hbox {keV}$ has been reported to be $\mu _{LT} = 0.24\,$cm$^2$/g. Based on the maximal dark-field signal obtainable before saturation

8 $$\begin{aligned} \Sigma _{max} = -\ln \left( \frac{\sigma _{\Sigma }(n, I_0, t)}{V_0}\right) = -\frac{1}{2}\ln \left( \frac{\pi }{nI_0e^{-\mu t}V_0^2}\right) = -\frac{1}{2}\left( \ln \left( \frac{\pi }{nI_0V_0^2}\right) + \ln \left( \frac{1}{e^{-\mu t}} \right) \right) = -\frac{1}{2}\left( \ln \left( \frac{\pi }{nI_0V_0^2}\right) + \mu t\right) , \end{aligned}$$

where $\sigma _{\Sigma }$ is the noise floor from where the visibility can not be detected anymore. With this equation we can define a sensitivity of the measurement as

9 $$\begin{aligned} \tau (I_0, V_0) = V_0\sqrt{nI_0}. \end{aligned}$$

The maximal dark-field measurable for a certain sensitivity given by Eq. (9) and thickness yields

10 $$\begin{aligned} \frac{\Sigma _{max}}{R(\xi )} = \Gamma = \ln \left( \frac{I_0}{I_1}\right) . \end{aligned}$$

Inserting $I_1 = I_0 e^{-\mu _{LT} \rho _L t_{max}}$ and solve for $t_{max}$ leads to

11 $$\begin{aligned} t_{max}(\xi ) = \frac{-\ln \left( \frac{\pi }{nI_0V_0^2}\right) }{(2R(\xi )+1)\mu _{LT}\rho _L} = \frac{-\ln \left( \frac{\pi }{\tau ^2}\right) }{(2R(\xi )+1)\mu _{LT}\rho _L}, \end{aligned}$$

where $t_{max}$ is the maximal sample thickness before saturation and $R(\xi )$ is the R-value of material in question at the autocorrelation length $\xi$.

**Figure 6:**
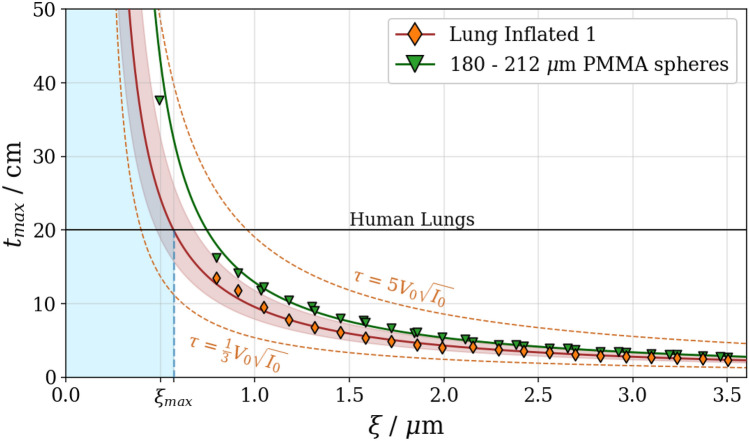
The maximal lung thickness possible to image at a given autocorrelation length without encountering saturation of the dark-field signal according to Eq. (11). Measurements indicated in orange were taken at $V_0 = 6 \%$ and $nI_0 = 80'000$ which represents our imaging parameters. The shaded region around the red line indicates the region of uncertainty due to the measurement and fitting accuracy. In green the 180–212 $\upmu \hbox {m}$ PMMA spheres are displayed which have one data point well beyond the 20 cm line that allows interpolation, while the lung data has to be extrapolated. Lines for different $\tau$ are indicated. The blue area indicates $\xi$ below ${0.57}\,\upmu \hbox {m}$, which allows to image healthy human lungs in an inflated state avoiding saturation effects.

Figure 6 shows the corresponding values of $t_{max}$ for the series ’Lung Inflated 1’ and the 180–212 $\upmu \hbox {m}$ PMMA spheres. $R(\xi )$ is thickness-independent for homogeneous samples without saturation. For the constant thickness measurements, it corresponds to a scaled graph of the dark-field. The dark-field extinction coefficient increases over the autocorrelation length from zero until the feature size of the diffusing sample is reached and is constant thereafter (shown by Gkoumas et al.^28^ in Fig. 4 or Strobl^26^ in Fig. 3b). The *R* values that were measured over our autocorrelation range yield a straight line as shown in Fig. S3 (Supplementary Information). This allows to interpolate the data with a first-degree polynomial. Due to the gradual approach of R($\theta$) to 0, the extrapolation of the polynomial leads to an upper bound. The exact slope provided in Fig. S3 corresponds to an effective energy of ${46}\,\hbox {keV}$ at ${70}\,\hbox {kVp}$ of a tungsten tube and may vary for different spectra.

The maximal traversed path for X-rays within lung tissue in a posterior-anterior imaging orientation in humans was estimated to be roughly $t_{max} = {20}\,\hbox {cm}$ based on open accessible human chest CT scans^50^ and is indicated in Fig. 6 by the black line. Pathologies only lead to a decrease in dark-field signal and as such operating close to $\xi _{max}$ is reasonable. The measurement sensitivity $\tau (I_0, V_0)$ allows to connect imaging time/dose and the visibility with the maximal thickness achievable at a certain autocorrelation length.

## Discussion

In this study a porcine lung inflation chamber is presented together with data of lung and PMMA microsphere dark-field signal over a wide range of the autocorrelation length. The aim was to provide data to the saturation limits of dark-field lung imaging and system parameters to prevent saturation from occurring.

We established the means to obtain, handle, inflate and image porcine lungs in a negative pressure container. Even though the porcine lungs had cuts and injuries from the slaughtering process the degree of inflation followed the applied pressure difference inside the vacuum chamber. As the lungs were contained in a closed container with almost no air circulation their surface did not dry and they stayed humid also over longer imaging sessions. While the system presented here reliably inflates the lungs and maintains a constant pressure comparable to *in vivo* conditions, it lacks distinct features present in the human body. Most notably the lack of a volume-confining structure acting against the lung’s expansion similar to the human rib-cage and its musculature as well as the lack of the heart and any circulatory effects.

The study supports findings^33,34^ that the thickness–corrected dark-field signal *R* of inflated porcine lungs is found to be similar to the one of PMMA spheres with a size range of 20–500 $\upmu \hbox {m}$ as shown in Fig. 5. For the comparison of lung structures with PMMA spheres a correction of the *R*-value by a factor 1.778 was necessary based on the inverted matter/air ratio, assuming average dense sphere packing. The exact value of this factor is not significant as it is used for qualitative comparisons only. As such, PMMA spheres are an easily obtainable suitable stand-in for lung samples in terms of the dark-field signal.

Gkoumas et al. reported that the dark-field signal of hard spheres stabilizes once $\xi = d$, where $\xi$ is the autocorrelation length and d is the diameter of the spheres^28^. This stabilizing effect, however, is not observable in our measurements, as the maximal autocorrelation length $\xi$ reached was ${5.2}\,\upmu \hbox {m}$ compared to the smallest imaged structure diameter of ${20}\,\upmu \hbox {m}$. In our case, the asymptotic tendency, best seen in the 20–27 $\upmu \hbox {m}$ PMMA spheres, towards a constant dark-field signal can clearly be attributed to signal saturation. To not misinterpret results it is important to attribute a level of saturation (as shown in Eq. (6) and Fig. 4) to each pixel of the measurements.

On the basis of our measurements a maximal sample thickness can be estimated for a given autocorrelation length. Assuming a maximal lung thickness in an anterior-posterior (AP) orientation of ${20}\,\hbox {cm}$, a maximal autocorrelation length of $\xi _{max} = {0.57}\,\upmu \hbox {m}$ results, as indicated in Fig. 6. For practical applications operating at the maximal level of detectable visibility loss is not recommended as saturation effects already influence the results before reaching the threshold. The crossing point of the lung data with a ${20}\,\hbox {cm}$ thickness could only be obtained through extrapolation. To provide an interpolated crossing point the closest PMMA spheres to the lung with sizes of ${180}-{212}\,\upmu \hbox {m}$ were displayed as well. The small container of these spheres allowed to obtain a measurement at lower autocorrelation length which provides a crossing point with the ${20}\,\hbox {cm}$ lung thickness estimation. The linearity of the *R* value is only given until saturation effects start to appear. For the shown ${180}-{212}\,\upmu \hbox {m}$ spheres this was not the case and the data points were therefore on a straight line.

Signal saturation in dark-field imaging is an expected effect that occurs once the noise level surpasses the visibility of the phase stepping curve. As dark-field imaging relies on the visibility loss caused by the sample due to small angle scattering, the imaging setup has to be designed such that it is capable of handling the intended sample without saturation. Maximising the visibility is an integral part of optimizing imaging systems and helps to reduce the saturation score as well as increasing the dose efficiency. In our setup the visibility of 6 $\%$ is much lower than in similar setups^22^. We attribute it to local defects of the absorption gratings, mainly due to the small bending radius of $\text {G}_0$^42^. The local change in the periodicity reduces the coherence downstream with a magnification of 17 on $\text {G}_2$ decreasing the visibility. While it is more challenging to reach low noise levels with low visibility, it does not impair the results in any other way. The sensitivity parameter $\tau$ from Eq. (9) allows to estimate the gain for optimizations in visibility or dose. For clinical applications the dose, or $I_0$, is to be minimized. Increasing the visibility allows to reduce the dose, but the saturation issue can not reasonably be solved by it. For a clinical lung imaging device, it is therefore necessary to use design parameters that put the autocorrelation length to a suitable value as shown in Fig. 6.

## Conclusion

The emergence of first systems for dark-field lung radiography and CT raises the question of the range of parameters applicable for clinical imaging, not unlike the choice of the energy in attenuation imaging. In particular the autocorrelation length is the defining parameter for the sensitivity of DF imaging systems. Furthermore, just as attenuation imaging is susceptible to a loss of signal due to photon starvation, a similar saturation effect is observed in DF imaging due to a complete loss of resolvable visibility. We find out that the autocorrelation length of the system is the crucial parameter for the clinical value it can provide. A lung imaging system should be designed to operate at as high autocorrelation lengths as the visibility and dose allow without encountering saturation effects.

We report on a measurement of the DF signal of inflated porcine lungs in a wide range of autocorrelation lengths with a Talbot-Lau interferometer. We have identified the saturation of the signal to be the most likely design constraint for a human-scale DF imaging systems, because of a combination of a large thickness of the object and low dose. This favours designs with low autocorrelation length, which we quantify in a simplified lung-only case. Further work should also consider the effect of the surrounding tissue on both attenuation and visibility reduction. As the position of the measured object in a Talbot-Lau interferometer changes the autocorrelation length, it allows to tune the sensitivity of the system to the measured object. For quantitative applications, this poses an uncertainty, as the extended object has no uniform autocorrelation length. For clinical applications this can be an option to fine tune the sensitivity on a per-patient basis.

The comparison of the experiment with microspheres also allows the conclusion that the dark-field contrast is induced by structures with a mean size of a few hundred micrometers, which corresponds to the alveolar structure of the lung. Microspheres also provide an attainable and reliable lung substitute for qualitative testing of dark-field lung scanners. Therefore, PMMA microspheres are an attractive option for dark-field phantoms.

Given the potential of DF as a new clinical imaging modality, we hope that the framework we propose will help designing future clinical DF radiography systems.

## Supplementary Information

Supplementary Information.

## Data Availability

The data and analysis pipeline are publicly available at ETH Research Collection (10.3929/ethz-b-000598251) and Renku (https://renkulab.io/projects/stamplab/autocorrelation-scan-of-porcine-lungs), respectively.
