# Supplementary material for: The choice of an autocorrelation length in dark-field lung imaging
Source: Sci Rep. 2023 Feb 15;13:2731. doi: 10.1038/s41598-023-29762-y (PMC9932147; doi:10.1038/s41598-023-29762-y)
Supplement: Supplementary file 1 — Supplementary Information. [file 41598_2023_29762_MOESM1_ESM.docx]

**The choice of an autocorrelation length in dark-field lung imaging**

Simon Spindler^1,2,*,+^, Dominik Etter^1,2,+^, Michał Rawlik^1,2^, Maxim Polikarpov^1,2^, Lucia
Romano^1,2^, Zhitian Shi^1,2^, Konstantins Jefimovs^1^, Zhentian Wang^1^, and Marco
Stampanoni^1,2^

^1^Swiss Light Source, Paul Scherrer Institute, 5232 Villigen, Switzerland
^2^Institute for Biomedical Engineering, ETH Z ̈urich, 8092 Z ̈urich, Switzerland
^*^simon.spindler@psi.ch
^+^these authors contributed equally to this work

**Supplementary Information**

**Beam hardening correction**

Using continuous spectra for X-ray imaging, like photon fields from X-ray tubes, will cause beam hardening artifacts due to the energy dependency of the attenuation coefficient. Low energies will be more attenuated than high energies, resulting in a shift towards a harder spectral distribution or higher mean photon energy. This leads to an underestimation of attenuation which becomes more prominent the thicker the sample is. The visibility decreases as well which leads to a dark-field signal even in the absence of micro-structures^1^.

The beam hardening correction was performed by measuring PMMA plates between 1 – 18 cm. For every detector pixel a polynomial of second degree was fitted for absorption while for dark field a polynomial of first degree was used as shown in Fig. S1. An input value (blue line in Fig. S1) was rescaled to the green line according to the corresponding attenuation coefficient of PMMA^2^ at 46 keV (0.257 cm^-1^), removing the spectral component from the attenuation. The dark-field used the thickness from the absorption as the input parameter for the linear dark-field fitting parameters. The result was subtracted from the dark field. Another advantage of this method is that damaged pixels can be identified and interpolated over.


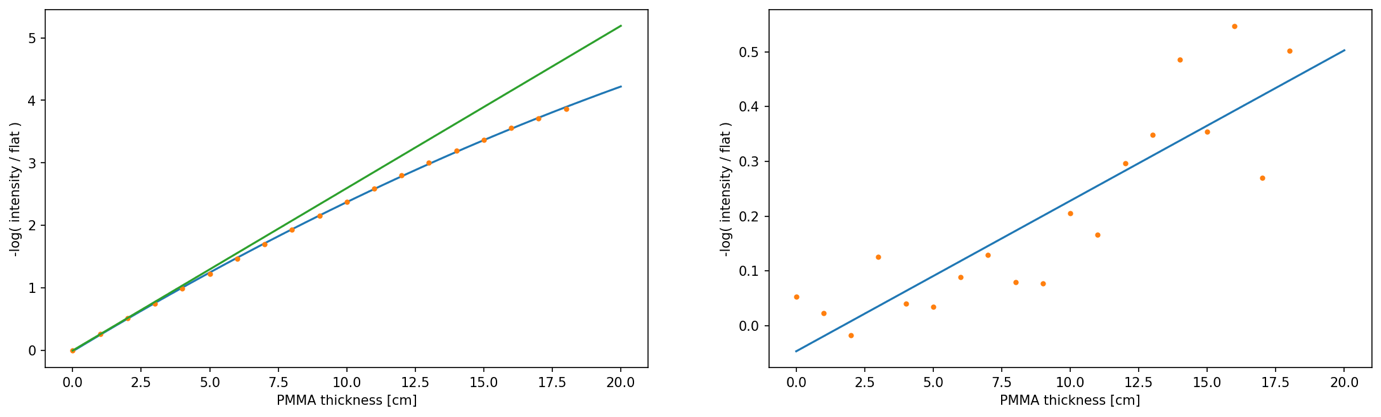


**Figure S1**. Measurements of PMMA plates between 1 – 18 cm. *Left*: The absorption measurements from a single pixel and the quadratic fit for the data points. *Right*: The dark-field signal of the PMMA plates and a linear fit for the data points.

**Lung at largest autocorrelation length
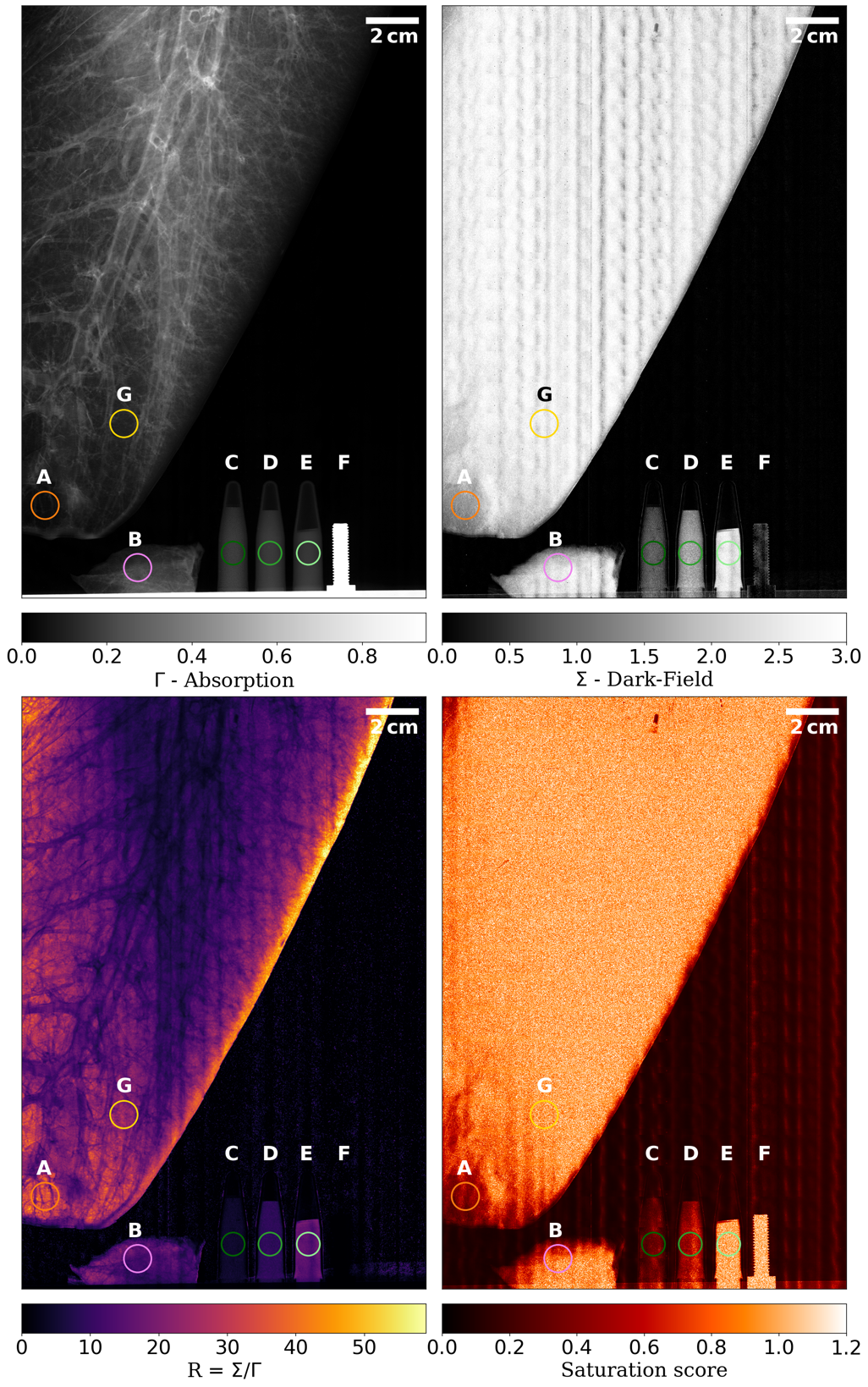
**

**Figure S2**. Analysed data from imaging done with sample shown in Fig. 5 at an autocorrelation length of $\xi$ = 3.6 $\mu$m. The displayed circles mark the regions of interest that are displayed in Fig. 5. *Top-left*: absorption, *top-right*: dark-field, *bottom-left*: R-value and *bottom-right*: saturation score. A) Lateral basal segment of the inflated left porcine lung ('Lung Inflated 1'). B) A piece of non-inflated porcine lung tissue. C, D, E) Eppendorf tubes containing PMMA spheres of 425 to 500, 180 to 212 and 20 to 27 $\mu$m diameter, respectively. F) A M4 steel screw (4 mm in diameter) as reference marker. G) thicker segment of inflated porcine lung ('Lung Inflated 2').

**R-value**

**
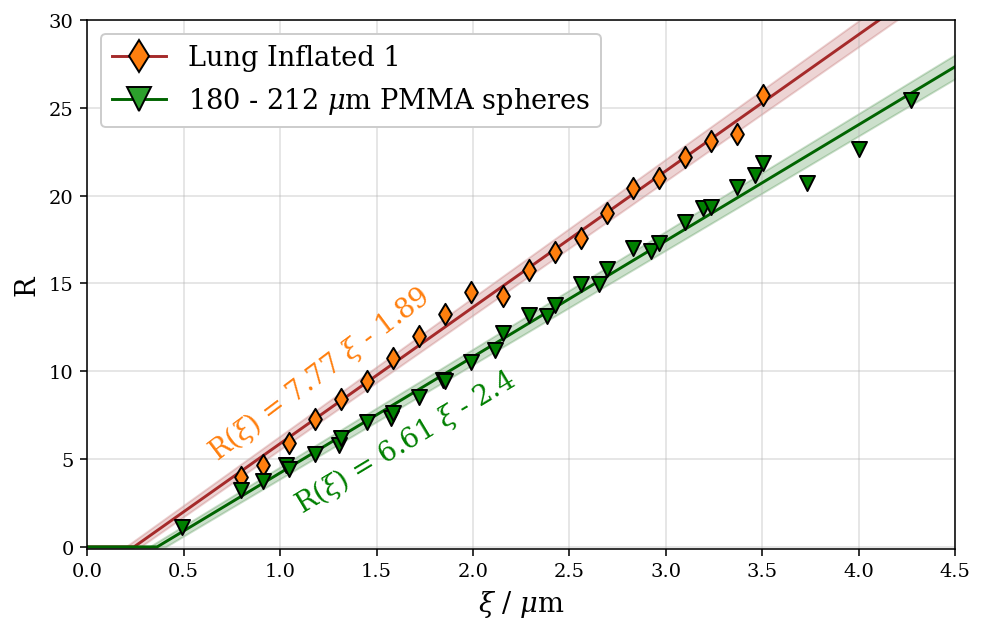
**

**Figure S3**. Plot of unsaturated R-value measurements over the autocorrelation length for the “Lung Inflated 1” and "180 – 212 $\mu$m PMMA spheres” (region of interest shown in Fig. 4). The data was fitted by a first-degree polynomial and is displayed with the ideal fit as well as the uncertainty. The R-value was set to zero for autocorrelation lengths where the fit of the R-value would yield negative values. These fits are used as an input for Eq. 11 which is shown in Fig. 6.

**References**

1. Yashiro, W., Vagovič, P. & Momose, A. Effect of beam hardening on a visibility-contrast image obtained by x-ray grating interferometry. Opt. Express 23, 23462–23471, DOI: 10.1364/OE.23.023462 (2015)
2. X-Ray Mass Attenuation Coefficients | NIST. https://www.nist.gov/pml/x-ray-mass-attenuation-coefficients, Accessed on 23.09.2022.
